# Effects of N-Acetylcysteine and Alpha-Ketoglutarate on OVCAR3 Ovarian Cancer Cells: Insights from Integrative Bioinformatics and Experimental Validation

**DOI:** 10.3390/cells15030281

**Published:** 2026-02-02

**Authors:** Yasaman Khamineh, Sanaz Panahi-Alanagh, Samaneh Zolghadri, Laleh Mavaddatiyan, Ireneusz Ryszkiel, Agata Stanek, Mahmood Talkhabi

**Affiliations:** 1 Department of Animal Sciences and Marine Biology, Faculty of Life Sciences and Biotechnology, Shahid Beheshti University, Tehran 1983969411, Iran; yasaman.khamine@gmail.com (Y.K.); sanazpanahialanagh@gmail.com (S.P.-A.); laleh.mavaddat99@gmail.com (L.M.); 2 Department of Biology, Jahrom Branch, Islamic Azad University, Jahrom 7414785318, Iran; z.jahromi@ut.ac.ir; 3 Department of Internal Medicine, Metabolic Diseases and Angiology, Faculty of Health Sciences in Katowice, Medical University of Silesia, Ziolowa 45/47 St., 40-635 Katowice, Poland; ryszkielirek@gmail.com

**Keywords:** ovarian cancer, N-acetylcysteine, alpha-ketoglutarate, network pharmacology, cell viability, apoptosis, cell migration

## Abstract

Ovarian cancer remains one of the leading causes of cancer-related mortality among women, underscoring the need for novel combination strategies that effectively inhibit tumor cell growth while limiting adverse effects. N-acetylcysteine (NAC) and alpha-ketoglutarate (AKG) are biologically active compounds with reported anticancer properties; however, their combined effects in ovarian cancer are not well characterized. In this study, we applied an integrative approach combining network pharmacology analysis with in vitro experiments to investigate the effects of NAC and AKG on OVCAR3 ovarian cancer cells. Common molecular targets of NAC and AKG were identified by intersecting predicted compound targets with ovarian cancer-associated genes, followed by protein–protein interaction network construction and Gene Ontology and Kyoto Encyclopedia of Genes and Genomes pathway enrichment analyses. Experimental validation assessed the effects of NAC and AKG, alone and in combination, on cell viability, apoptosis, migration, and clonogenic capacity. Network analysis identified 70 shared target genes enriched in pathways related to apoptosis, cellular stress responses, and cell migration. In vitro experiments demonstrated that combined treatment with NAC (10 mM) and AKG (100 µM) significantly reduced cell viability, increased apoptotic cell death, and markedly suppressed cell migration and colony formation compared with single-agent treatments. Overall, these findings indicate that the combination of NAC and AKG exerts enhanced inhibitory effects on ovarian cancer cell growth and motility in vitro.

## 1. Introduction

Ovarian cancer is one of the most prevalent cancers in women and is associated with a very high mortality rate, with a global five-year survival of approximately 46%, making it the deadliest gynecological malignancy [1,2,3]. The disease comprises distinct histological subtypes that differ substantially in incidence, site of origin, pathogenesis, gene expression patterns, molecular alterations, and prognosis [4,5]. Menopause, family history, obesity, and endometriosis are among the established risk factors for ovarian cancer [6,7]. The high mortality of this disease largely results from late diagnosis, which is attributable to the lack of specific early clinical symptoms and effective screening strategies [8]. Consequently, most patients are diagnosed at an advanced stage, frequently with metastatic disease [9]. These challenges highlight the urgent need for improved preventive, diagnostic, and therapeutic strategies, including combination approaches that target multiple disease-relevant processes while preserving ovarian function and fertility [5,10].

Alpha-ketoglutarate (AKG), also known as 2-oxoglutarate (2-OG), is a central intermediate of the tricarboxylic acid (TCA) cycle and plays a key role in cellular metabolism [11,12]. Depending on cellular context and concentration, AKG participates in diverse biological processes, including energy metabolism, redox homeostasis, signal transduction, and regulation of gene expression [12,13]. As a precursor of glutamate and glutamine, AKG supports protein synthesis and serves as an important metabolic substrate for rapidly proliferating cells [14,15]. AKG metabolism is closely linked to glutamine utilization, and previous studies have shown that cancer cells can exploit AKG–glutamine interconversion to support biosynthetic and energetic demands [16,17]. In parallel, accumulating evidence suggests that AKG may exert antitumor effects in certain contexts, including modulation of the tumor microenvironment, enhancement of immune responses, and suppression of cancer cell proliferation, migration, and metastatic potential [11,18]. AKG has also been implicated in the regulation of signaling pathways associated with cellular stress responses and epigenetic remodeling, such as MAPK activity, prolyl hydroxylase–dependent processes, and DNA demethylation mediated by ten-eleven translocation (TET) enzymes [18,19,20].

N-acetylcysteine (NAC) is a thiol-containing compound and a well-established precursor of intracellular glutathione. Clinically, NAC has long been used as a mucolytic agent and as an antidote for acetaminophen overdose [21,22]. Beyond these applications, NAC has been reported to influence mitochondrial function, oxidative stress, and inflammatory responses [21,23]. Many of its biological effects are attributed to modulation of cellular redox balance through detoxification of reactive oxygen species, enhancement of glutathione synthesis, and activation of antioxidant defense pathways such as NRF2 [21]. In cancer models, NAC has been shown to exert context-dependent effects, including inhibition of proliferation, migration, and survival in several tumor types [24,25]. These findings suggest that NAC may influence multiple cellular processes relevant to tumor progression.

Given their distinct yet potentially complementary biological activities, the combination of NAC and AKG may provide a multifaceted approach to modulating ovarian cancer cell behavior. However, the combined effects of these compounds in ovarian cancer models remain insufficiently characterized.

**Figure 1:**
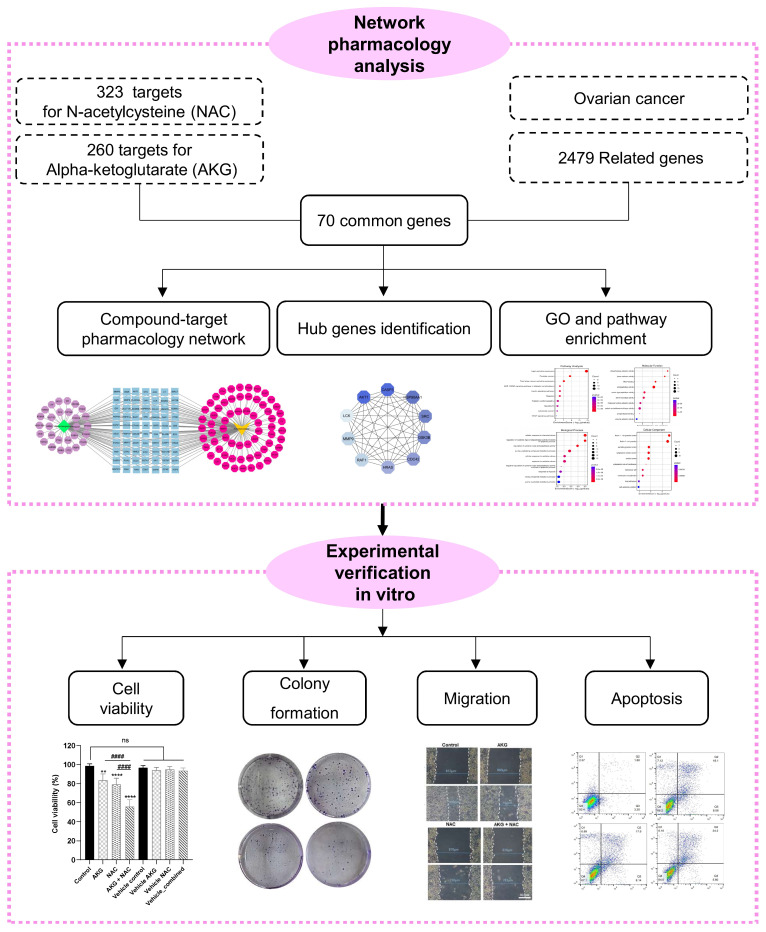
Research flowchart. Schematic workflow of the study. Network pharmacology analysis was integrated with in vitro experiments to investigate the effects of NAC and AKG on OVCAR3 ovarian cancer cells. Bioinformatics approaches were used to identify common molecular targets and enriched biological pathways. Experimental verification focused on phenotypic outcomes, including cell viability, apoptotic cell death, migration, and clonogenic capacity.

Therefore, the present study aimed to investigate the combined effects of NAC and AKG on the OVCAR3 ovarian cancer cell line using an integrative strategy that combines network pharmacology analysis with in vitro experimental assays. Network pharmacology was applied to identify common molecular targets and enriched biological pathways associated with NAC, AKG, and ovarian cancer. Experimental validation focused on phenotypic outcomes, including cell viability, apoptotic cell death, migration, and clonogenic capacity. Exploratory molecular observations are provided in the Supplementary Materials to offer contextual support for the observed cellular effects. Together, these findings provide a framework for future studies exploring the therapeutic potential of NAC and AKG in ovarian cancer. The overall workflow of the study is illustrated in Figure 1.

## 2. Materials and Methods

### 2.1. Network Pharmacology Analysis

#### 2.1.1. Identification of Common Genes Related to AKG, NAC, and Ovarian Cancer

The targets for NAC and AKG in *Homo sapiens* were obtained from SwissTargetPrediction and PharmMapper databases. SwissTargetPrediction is a widely used cheminformatics platform that predicts potential small molecule–protein interactions based on the high-dimensional structural similarity of 2D and 3D models to known bioactive compounds (http://www.swisstargetprediction.ch/, accessed on 2 July 2025). PharmMapper is a reverse pharmacophore mapping server that aligns query compounds with a comprehensive library of pharmacophoric models derived from targetBank, BindingDB, DrugBank, and Potential Drug Target Database (PDTD) to prioritize potential targets (https://www.lilab-ecust.cn/pharmmapper/, accessed on 2 July 2025). The genes associated with ovarian cancer were extracted from the GeneCards database (https://www.genecards.org/, accessed on 8 July 2025) (with a Relevance score > 10, Gifts > 40). GeneCards provides genomic, transcriptomic, proteomic, genetic, clinical, and functional information, with literature sources that have applied filters to ensure that only high-confidence, disease-relevant genes are included. UniProtKB (https://www.uniprot.org, accessed on 10 July 2025) was used to convert protein names into gene names under “*Homo sapiens*” conditions. Duplicate targets were removed from the dataset. Finally, common genes were intersected using a Venn diagram (https://molbiotools.com, accessed on 10 July 2025).

#### 2.1.2. Compound–Target Pharmacology Network Construction

A protein–protein interaction (PPI) network comprising the common genes was generated using the STRING database (https://string-db.org, accessed on 12 July 2025) and subsequently visualized with Cytoscape (version 3.10.1). Hub genes were identified with the cytoscape-cytoHubba plugin using the Maximal Clique Centrality (MCC) algorithm. The MCC method ranks each node according to the number and size of maximal cliques in which it participates, giving higher scores to nodes that are highly interconnected within dense clusters. The top 10 highest-scoring genes were selected as hub genes and illustrated in Cytoscape. In addition, to avoid dependence on a single metric, we also computed three complementary centrality measures (Degree, Betweenness, and Closeness). To assess the robustness of hub selection, we performed analysis by filtering the PPI edges using multiple STRING interaction-score thresholds (0.4, 0.7, and 0.9) and re-running cytoHubba hub ranking on each resulting subnetwork. Nodes with degree zero after filtering were removed prior to hub ranking. Additional exploratory transcriptomic analyses and protein expression assessments related to selected network genes with the highest ranking scores were performed using public ovarian cancer datasets and the OVCAR3 cell model; methodological details are provided in the Supplementary Materials (Sections S1.1 and S1.2).

#### 2.1.3. Enrichment Analysis

Gene Ontology (GO) and KEGG (Kyoto Encyclopedia of Genes and Genomes) pathway enrichment analyses of common targets of AKG and NAC against ovarian cancer-related genes were conducted using the Enrichr (https://maayanlab.cloud/Enrichr/, accessed on 12 July 2025) web tool and visualized using the SRplot (https://www.bioinformatics.com.cn/, accessed on 12 July 2025) online tool.

### 2.2. Experimental Validation

To validate the bioinformatics predictions, experimental assays were performed using the human ovarian cancer cell line OVCAR3.

#### 2.2.1. Preparation of NAC and AKG Stock Solutions and Vehicle Controls

NAC and AKG solutions were freshly prepared using sterile distilled water as the solvent. For NAC, 1.63 g of NAC powder (Bio Basic Inc., Markham, ON, Canada, Lot No. NB561830) was dissolved in 10 mL sterile distilled water to prepare a 1 M stock solution, which was filtered through a 0.22 µm membrane. For preparation of the working solution, 100 µL of the NAC stock was added, and the volume was adjusted to 10 mL with culture medium, resulting in a final concentration of 10 mM. The corresponding vehicle was prepared by adding 100 µL of sterile distilled water and adjusting the volume to 10 mL with medium (1% (*v*/*v*)). The AKG stock solution was prepared by dissolving 7.3 mg of AKG (Bio Basic Inc., Markham, ON, Canada, Lot No. PB043010) in 10 mL of sterile distilled water to obtain a 5 mM solution, which was subsequently sterilized by filtration. To prepare the 100 µM working solution, 200 µL of the AKG stock was added, and the volume was brought to 10 mL with culture medium (2% (*v*/*v*)). The vehicle was prepared by adding 200 µL of sterile distilled water and adjusting the volume to 10 mL. For the combined treatment, 100 µL of the NAC stock and 200 µL of the AKG stock were added, and the final volume was adjusted to 10 mL with complete medium (3% (*v*/*v*)). The matched vehicle_combined was prepared by adding 300 µL of sterile distilled water and bringing the volume to 10 mL.

#### 2.2.2. pH Adjustment of Treatment and Vehicle Media

After preparation, all media (treatments and vehicles) were equilibrated for 30 min at 37 °C in 5% CO_2_, and the pH was measured using a calibrated microelectrode pH meter. Because the combination treatment received the highest total amount of added compounds, the pH of all treatments and vehicle preparations was adjusted to match that of the combination group. This adjustment was achieved using a very small volume of 0.1 N HCl (<10 microliter per 10 mL, stepwise microliter-volume additions). For all assays, the vehicle_combined (300 µL sterile distilled water per 10 mL complete medium, pH-adjusted to match the NAC + AKG combination) was used as the common vehicle for all treatment groups. This approach ensured that all groups entered the experiments under identical physicochemical conditions.

#### 2.2.3. Cell Culture and Treatments

The human ovarian cancer cell line OVCAR3 was cultured in Roswell Park Memorial Institute medium (RPMI-1640) (Bioidea, Tehran, Iran) supplemented with 10% fetal bovine serum (FBS) (Gibco, Grand Island, NY, USA) and 1% penicillin–streptomycin (Gibco, Grand Island, NY, USA). They were maintained at 37 °C in the incubator with 5% CO_2_ and 95% humidity. The culture medium was replaced every three days.

#### 2.2.4. MTT Assay

Cell viability was evaluated using the 3-(4,5-dimethylthiazol-2-yl)-2,5 diphenyltetrazolium bromide (MTT) (Sigma-Aldrich, St. Louis, MO, USA) assay. OVCAR3 cells (5 × 10^3^ cells/well) were seeded in a 96-well plate, incubated for 24 h, and allowed to attach. Following a 24 h treatment period under various concentrations of AKG (80, 100, 120, 140, and 160 µM) and NAC (5, 10, 15, 20, 25, 30, and 40 mM), a 0.5 mg/mL solution of MTT was added to each well and incubated for 3 h. Afterwards, the medium containing MTT was discarded, and 100 μL of dimethylsulfoxide (DMSO) (Sigma-Aldrich, St. Louis, MO, USA) was added to each well. The absorbance was then measured at 570 nm using a microplate reader (Bio-Tek, Winooski, VT, USA) [26]. For each treatment group, a dedicated vehicle control matched in solvent volume and fully pH-adjusted was incorporated, and cell viability was measured under identical experimental conditions. Each experimental condition was repeated three times for each group. The percentage of cell viability was calculated using the following formula:

$$Cell\;viability=\frac{Absorbance(Test)-Absorbance(Blank)}{Absorbance(Vehicle)-Absorbance(Blank)}\times 100$$

#### 2.2.5. Synergistic Analysis

To formally assess the interaction between NAC and AKG, combination effects on cell viability were quantified using two reference models: Bliss independence and the Highest Single Agent (HSA), based on 24 h MTT viability data from a two-dimensional NAC–AKG concentration matrix normalized to the combined vehicle control; using viability values V(%), $V_{\mathrm{Bliss}}=(V_{\mathrm{NAC}}\times V_{\mathrm{AKG}})/100$ and $V_{\mathrm{HSA}}=min(V_{\mathrm{NAC}},V_{\mathrm{AKG}})$, and interaction was reported as $\Delta =V_{\mathrm{expected}}-V_{\mathrm{observed}}$, where Δ>0 indicates synergy, Δ≈0 indicates additivity, and Δ<0 indicates antagonism.

#### 2.2.6. Annexin V/PI Assay

The apoptotic effects of NAC and AKG on OVCAR3 cells were examined via the Annexin V detection kit (Invitrogen, Carlsbad, CA, USA) using flow cytometry. OVCAR3 cells were seeded in 6-well culture plates at a density of 2 × 10^5^ cells per well. The cells were treated with 10 mM NAC, 100 µM AKG, or a combination of both for 24 h at 37 °C in a 5% CO_2_ incubator. After treatment, the cells were collected and fixed with pre-cooled 4% paraformaldehyde. The cells were then centrifuged at 2000 rpm for 5 min and washed twice with PBS. The resulting cell pellets were resuspended and stained with 5 μL of Propidium Iodide (PI) and Annexin V in 500 μL of Annexin Binding Buffer (ABB), according to the guidelines provided by the manufacturer. Following staining, the cells were incubated in the dark for 20 min before being analyzed via flow cytometry using a BD FACSCalibur flow cytometer (Biosciences, San Jose, CA, USA).

#### 2.2.7. Scratch Assay

To validate the enrichment analysis results and the migratory capacity of OVCAR3 cells, a scratch assay was conducted. This assay evaluated the effects of NAC, AKG, and a combination of both on the migration rate of OVCAR3 cells. OVCAR3 cells were cultured in 6-well plates at a density of 2 × 10^5^ cells per well. When the cells reached 85–90% confluence, they were treated with 10 μM of Mitomycin C (Sigma-Aldrich, St. Louis, MO, USA) solution for 3 h to prevent cell proliferation. Subsequently, a wound was created in each well using a 20 μL micropipette tip held at a 45° angle. The wells were washed with PBS solution to remove the detached cells. Then, fresh cell culture medium was added to the wells and treated with 100 µM AKG, 10 mM NAC, and a combination of both. Then, the plates were incubated at 37 °C in a humid atmosphere containing 5% CO_2_. A specific point along the scratch was selected, and the distance between the two edges of the wound was measured immediately after the wound was created (T0) and 24 h after (T24) using Mosaic software (version 2.4.0) and an inverted microscope (Zeiss, Oberkochen, Germany) [27]. Finally, the wound closure percentage was analyzed by comparing the changes in scratch diameter among the treated groups using the following formula:

$$Wound\;closure\;(\%)=\frac{T0-T24}{T0}\times 100$$

#### 2.2.8. Colony Formation Assay

OVCAR3 cells were seeded into 6-well plates at a density of 200 cells per well and incubated overnight at 37 °C. After 24 h, the cells were treated with 100 μM AKG, 10 mM NAC, and a combination of both for 10 days. The culture medium containing treatment agents was refreshed every 3 days. At the end of the 10 days, the cells were washed with PBS and fixed with paraformaldehyde (Merck, Darmstadt, Germany) for 15 min. They were then stained using 1% crystal violet solution for 20 min, washed with PBS, and the colonies were counted [28].

#### 2.2.9. Statistical Analysis

The experimental data are presented as mean ± standard deviation (SD). Statistical analyses were performed using GraphPad Prism software (version 10.6.1). All experiments were conducted using three independent biological replicates with five technical replicates per condition, except for Western blotting and flow cytometry, which were performed as exploratory single-run experiments and were therefore not subjected to statistical analysis. Comparisons among multiple groups were carried out using one-way analysis of variance (ANOVA). A *p*-value < 0.05 was considered statistically significant.

## 3. Results

### 3.1. Identification of Targets of NAC and AKG in Ovarian Cancer

**Figure 2:**
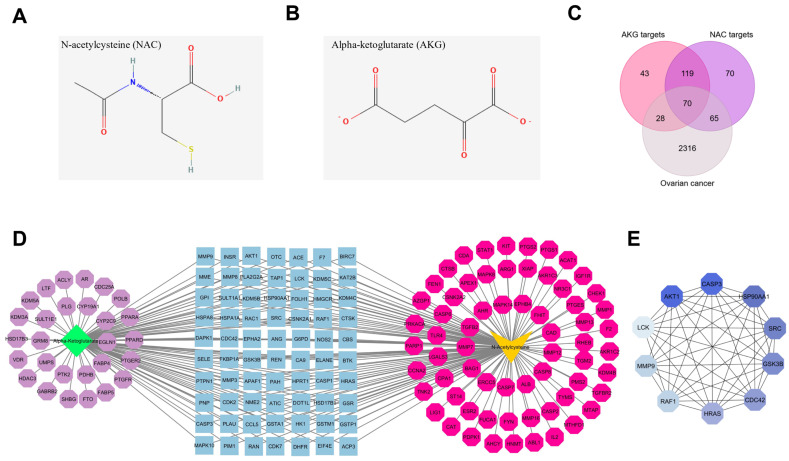
Network pharmacology analysis. (**A**,**B**) 2D structure of NAC and AKG. (**C**) Venn diagram indicating common genes between the targets of NAC, AKG, and ovarian cancer-related genes. (**D**) Compound–target pharmacology network: Purple and pink circles represent targets of NAC and AKG, respectively, while blue circles represent common targets of both NAC and AKG in ovarian cancer. (**E**) The top 10 hub genes identified using the MCC algorithm. Nodes with darker colors represent higher MCC scores and thus greater centrality within the PPI network.

The PharmMapper and SwissTargetPrediction databases identified 323 potential targets for N-acetylcysteine (NAC) and 260 potential targets for alpha-ketoglutarate (AKG) based on their two-dimensional chemical structures (Figure 2A,B; Supplementary Table S1). Ovarian cancer–related genes were retrieved from the GeneCards database. To ensure high confidence, only genes with a relevance score > 10 and a GIFtS score > 40 were included, yielding 2479 ovarian cancer-associated genes.

By intersecting the predicted targets of NAC and AKG with ovarian cancer–related genes, 70 common genes were identified using a Venn diagram approach (Figure 2C). A compound–target interaction network integrating NAC, AKG, and the shared targets was subsequently constructed using Cytoscape software (Figure 2D). In this network, nodes centrally located within the protein–protein interaction (PPI) structure were influenced by both compounds, whereas peripheral nodes were associated predominantly with a single compound.

Hub gene analysis using the MCC algorithm identified 10 highly connected genes, including *AKT1*, *CASP3*, *HSP90AA1*, *SRC*, *GSK3B*, *CDC42*, *HRAS*, *RAF1*, *MMP9*, and *LCK* (Table 1, Figure 2E). Hub prioritization proved robust across different STRING confidence thresholds (0.4, 0.7, and 0.9) and alternative centrality measures (Degree, Betweenness, and Closeness), with *AKT1* and *CASP3* consistently ranking among the highest-scoring nodes (Supplementary Table S2). Based on their network centrality, *AKT1* and *CASP3* were selected for exploratory transcriptomic and protein-level assessment, with all corresponding analyses reported in the Supplementary Materials (Sections S2.1 and S2.2; Figures S1 and S2).

### 3.2. GO and KEGG Pathway Enrichment Analysis

**Figure 3:**
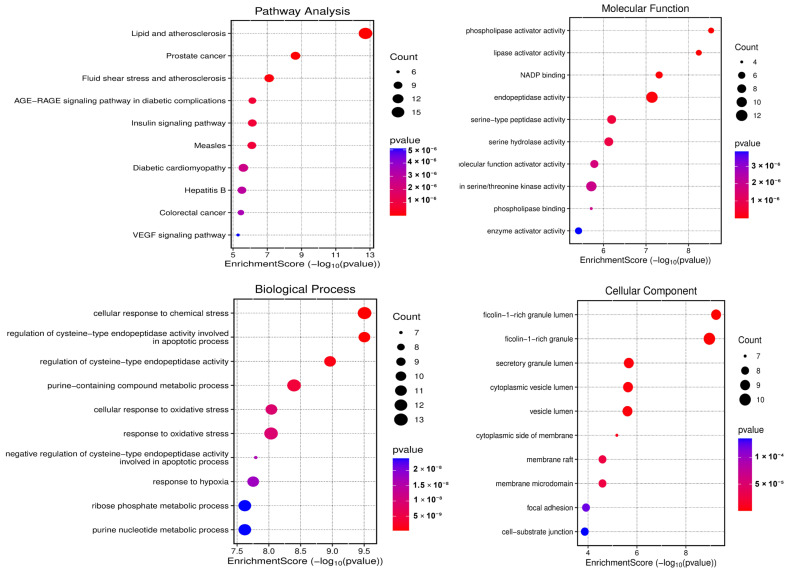
Functional enrichment of the 70 common targets of NAC, AKG, and ovarian cancer-related genes.

KEGG enrichment analysis showed that 70 common genes are highly enriched in the lipid and atherosclerosis, cancer-related, and fluid shear stress pathways, along with the AGE–RAGE signaling pathway in diabetic complications. GO enrichment analysis revealed that these common genes are significantly involved in improving several molecular functions (MFs), including phospholipase activator activity, lipase activator activity, NADP binding, and endopeptidase activity. Additionally, they are involved in various biological processes (BPs), including cellular response to chemical stress, cysteine-type endopeptidase activity involved in apoptosis, purine-containing compound metabolism, and cellular response to oxidative stress. In terms of cellular components (CCs), the common genes are mainly enriched in secretory/vesicle lumens (notably the ficolin-1-rich granule lumen), membrane microdomains, and adhesion sites (Figure 3).

### 3.3. In Vitro Validation

Based on the bioinformatics results, OVCAR3 cells were used as the in vitro model to verify the in silico predictions.

#### 3.3.1. NAC and AKG Reduced OVCAR3 Cell Viability

**Figure 4:**
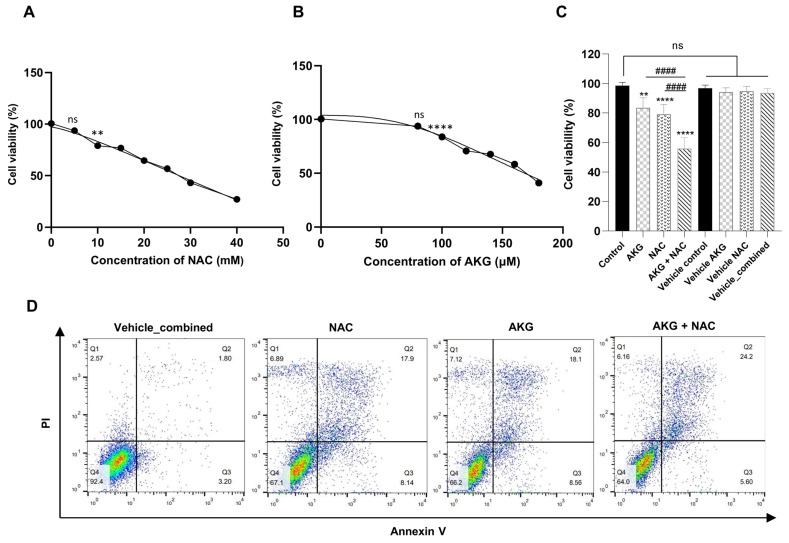
Effect of NAC and AKG on cell viability and apoptosis in OVCAR3 cells. (**A**,**B**) Cell viability of OVCAR3 cells treated with increasing concentrations of NAC (5–40 mM) and AKG (80–160 µM) for 24 h, assessed via MTT assay. (**C**) Cell viability of OVCAR3 cells in the treatment groups (10 mM NAC, 100 µM AKG, or their combination) and the volume-matched, pH-adjusted vehicle control. (**D**) Flow cytometry analysis of apoptosis and necrosis following treatment with 10 mM NAC, 100 µM AKG, or their combination. Quadrants indicate Q1: necrotic, Q2: late apoptotic, Q3: early apoptotic, and Q4: viable cell populations. MTT data (**A**–**C**) are presented as mean ± SD from three independent biological replicates (*n* = 3). Statistical analysis for (**A**–**C**) was performed using one-way ANOVA; ** *p* < 0.01 and **** *p* < 0.0001 vs. vehicle-combined; #### *p* < 0.0001 vs. single-compound treatments; ns = non-significant. Flow cytometry data (**D**) are from a single representative experiment (*n* = 1); therefore, no inferential statistics were applied to panel (**D**).

The MTT assay showed that the viability of OVCAR3 cells treated with NAC and AKG alone was significantly reduced in a dose-dependent manner after 24 h of treatment (Figure 4A,B). Specifically, treatment with 10 mM NAC decreased cell viability to 79.19%, whereas 100 µM AKG reduced viability to 83.61%. Notably, the combined treatment of 10 mM NAC and 100 µM AKG exerted a markedly stronger inhibitory effect (*p* < 0.0001), reducing cell viability to 55.93%, which was significantly lower than either single-agent treatment (*p* < 0.0001) (Figure 4C).

In addition, although there was a small difference in cell viability between the control group (98.6%) and the pH-matched vehicle_combined (96.2%), this difference was not statistically significant. Importantly, there were no significant changes in the cell viability among any of the vehicle groups, nor between these groups and the control. Consequently, the pH-standardized vehicle, adjusted to match the combination treatment, was used as the reference control in all subsequent experiments to ensure maximal accuracy and consistency across treatment conditions. Henceforth, this group will be referred to as the vehicle-combined group in all subsequent steps of the study.

#### 3.3.2. NAC and AKG Synergistically Reduced Cell Viability

At the selected dose pair (10 mM NAC and 100 µM AKG), single-agent viability was 79.19% for NAC and 83.61% for AKG, while the observed combination viability was 55.93%; under Bliss independence, the expected viability was $V_{\mathrm{Bliss}}=(79.19\times 83.61)/100=66.21\%$, giving $\Delta \mathrm{Bliss}=V_{\mathrm{Bliss}}-V_{\mathrm{obs}}=66.21-55.93=+10.28$%, and under the HSA model, the expected viability was $V_{\mathrm{HSA}}=\min\left(79.19,83.61\right)=79.19\%$, giving $\Delta \mathrm{HSA}=V_{\mathrm{HSA}}-V_{\mathrm{obs}}=79.19-55.93=+23.26$%. Collectively, the observed combination viability is lower than expected (Δ values > 0), indicating that the NAC and AKG combination reduces viability more than predicted by either Bliss independence or HSA at this dose pair (Supplementary Table S3).

#### 3.3.3. NAC and AKG Induced Apoptosis in OVCAR3 Cells

Apoptosis detection using flow cytometry analysis indicated that the percentage of apoptotic cells increased in groups treated with a combination of NAC and AKG. In the vehicle-combined group, the overall apoptosis rate was 5%, comprising 3.2% early apoptosis and 1.8% late apoptosis, with necrosis observed at 2.57%. Treatment with NAC increased the apoptosis rate to 26.04%, which included 8.14% early apoptosis and 17.9% late apoptosis, while necrosis rose to 6.89%. Similarly, treatment with AKG elevated the apoptosis rate to 26.66%, with 8.56% early apoptosis and 18.1% late apoptosis, and necrosis increased to 7.12%. Notably, the combined treatment of 10 mM NAC and 100 µM AKG further enhanced the apoptosis rate to 29.8%, consisting of 5.6% early apoptosis and 24.2% late apoptosis, while necrosis decreased to 6.16%. This combined treatment yielded a higher apoptosis rate than either compound alone (Figure 4D).

#### 3.3.4. NAC and AKG Inhibited Cell Migration in OVCAR3 Cells

**Figure 5:**
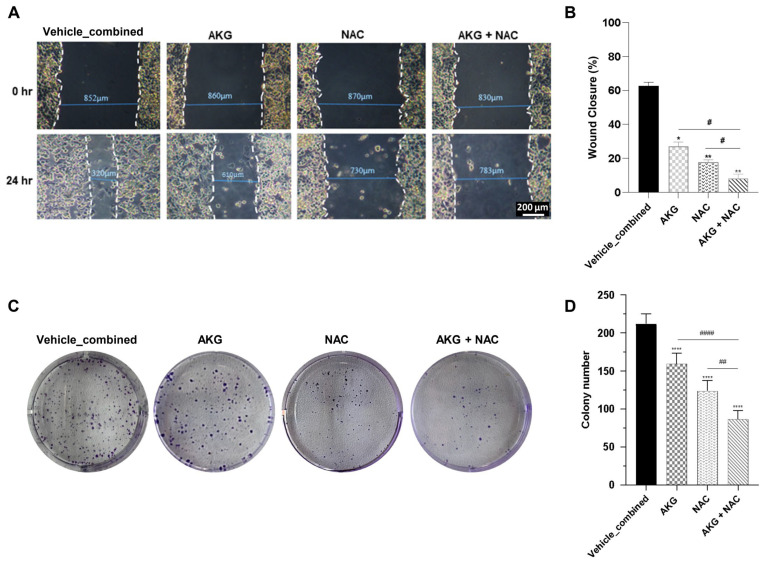
The effects of NAC and AKG on cell migration and clonogenic potential of OVCAR3 cells. (**A**) Representative microscopic images of OVCAR3 cell migration with the treatment of NAC, AKG, and a combination of both at T0 and T24 after treatment. (**B**) Quantitative analysis of the migration area indicated a percentage of wound closure. (**C**) Representative images of colony formation inhibition by NAC, AKG, and a combination of both. (**D**) Quantitative analysis of colony numbers. Statistical analysis for (**B**,**D**) was performed using one-way ANOVA; * *p* < 0.05, ** *p* < 0.01, **** *p* < 0.0001 compared to the vehicle-combined group; # *p* < 0.05, ## *p* < 0.01, and #### *p* < 0.0001 compared to the single-compound treatments. Data are represented as mean ± SD (*n* = 3).

The scratch assay revealed that approximately 60% of the wound was closed in the vehicle-combined group, indicating significant cell migration. Treatment with NAC alone reduced wound closure to approximately 16%, while AKG alone decreased it to around 29%. However, when NAC and AKG were used in combination, wound closure was reduced to only about 6%, showing a markedly greater inhibition of cell migration compared to either treatment alone (*p* < 0.05) (Figure 5A,B).

#### 3.3.5. NAC and AKG Suppressed Colony Formation of OVCAR3 Cells

The colony formation assay results showed a significant reduction in the number of colonies formed by OVCAR3 cancer cells when treated with the combination of NAC and AKG. In the vehicle-combined group, the number of colonies was approximately 200. Treatment with NAC alone reduced the colony number to approximately 120, while AKG lowered it to around 150. Notably, the combination of NAC and AKG further decreased the colony number to about 75, demonstrating a stronger inhibitory effect compared to each compound alone (*p* < 0.0001) (Figure 5C,D).

## 4. Discussion

NAC has recently been investigated as an anticancer agent, functioning either as a standalone treatment or as an adjunct therapy to suppress cell proliferation in various types of cancer [24,45,46,47]. In addition, AKG plays a pivotal role in a range of physiological processes, including cellular energy metabolism, amino acid/protein synthesis, cell death regulation, oxidative stress mitigation, and modulation of cancer cell behaviors [11,48]. Importantly, prior work suggests that the effects of NAC and AKG can be context dependent, varying with cancer type, genetic background, and experimental conditions. Numerous studies have shown that NAC effectively inhibits the viability, invasion, and migration of various cancer cells, including pancreatic [49], liver [50], breast [51], gastric [52], and glioblastoma cells [45]. Similarly, several studies have illustrated the anti-tumor effects of AKG and its ability to suppress the proliferation of cancer cells like colon adenocarcinoma cell lines [53], renal cell carcinoma (RCC) [54], osteosarcoma (OS) cells, and pancreatic ductal adenocarcinoma [19]. Therefore, the combination of AKG and NAC, two natural compounds with distinct biological activities, may represent an experimental strategy worth exploring in OVCAR3 cells. OVCAR3 is one of the most widely studied ovarian cancer cell lines and a representative model of high-grade serous ovarian cancer (HGSOC), the most common epithelial ovarian cancer subtype. It carries *TP53* loss and extensive copy number alterations, hallmark features of HGSOC), and has been included in large-scale genomic and drug-sensitivity studies, with numerous publications supporting its value as a preclinical model [55].

In this study, we used an integrative network pharmacology and in vitro approach to investigate cellular responses and associated molecular features related to treatment with 10 mM NAC and 100 µM AKG in OVCAR3 cells. A study conducted on Saos-2 and HOS osteosarcoma cell lines demonstrated that AKG reduced cell viability in a concentration-dependent manner by inhibiting cell cycle progression at the G1 phase. This effect was associated with a decreased expression of cyclin D1, a key regulator of the G1 to S phase transition, and an increased expression of the cyclin-dependent kinase inhibitor *p21Waf1/Cip1*, ultimately leading to cell cycle arrest and the inhibition of DNA replication [19]. In addition, high concentrations of NAC exhibit dose-dependent antiproliferative effects on OVCAR3 cells [56]. Another study demonstrated that a 1 h pretreatment of oral cancer cells with NAC and Burmannic acid (BURA) further reduced their viability [57]. Taken together, the enhanced effect of the combined AKG and NAC treatment observed in the MTT assay may be explained by prior reports describing antiproliferative properties of each compound across different cancer models. However, an important translational limitation is that the in vitro concentrations selected may not directly reflect physiologically or clinically achievable systemic exposures; therefore, the present findings should be interpreted primarily as proof-of-concept mechanistic evidence.

Bioinformatic analysis in this study identified 70 common genes shared between AKG, NAC targets, and ovarian cancer, highlighting potential shared biological processes relevant to ovarian cancer cell behavior. GO biological process analysis highlighted enrichment of cysteine-type endopeptidase activity, a process broadly associated with apoptosis and stress responses in cancer cells, where cysteine proteases (such as caspases and cathepsins) drive cell death, notably through caspase-3-mediated substrate cleavage [58,59]. Previous studies have reported that NAC can modulate apoptotic signaling, including mitochondrial-associated pathways. It has been reported that NAC can induce apoptosis through the mitochondrial pathway by altering the balance of pro- and anti-apoptotic Bcl-2 family proteins, promoting cytochrome c release, and activating caspase-9 and caspase-3 in H9c2 cells [60]. The apoptotic pathway is regulated by the BCL-2 family, which includes pro-survival proteins and pro-apoptotic members that promote programmed cell death. Pro-apoptotic BCL-2 family members are divided into two groups: the multi–BH domain effectors (*BAX*, *BAK*) that execute apoptosis, and the BH3-only initiator proteins (e.g., BID, BAD) that trigger the apoptotic process. In nutrient/growth-factor-rich conditions, pro-survival BCL-2 proteins inhibit BAX/BAK. Death signals upregulate BH3-only proteins, which antagonize pro-survival proteins and release BAX/BAK proteins. Activated BAX/BAK permeabilize the mitochondrial outer membrane, releasing cytochrome c. Cytochrome c forms the apoptosome with Apaf-1 and procaspase-9, activating caspase-9. Caspase-9 then activates executioner caspases (caspase-3, caspase-7), leading to cell death [61,62]. These well-characterized apoptotic cascades provide a biological framework that may help contextualize apoptosis-related phenotypes observed in the present study.

Moreover, alterations in AKG metabolism have been reported to influence mitochondrial function and cellular stress responses, which may intersect with cancer cell survival programs [63]. The cellular response to chemical stress pathway appears to be a central vulnerability in ovarian cancer. It links oxidative stress, inflammatory signaling, and hypoxic adaptation—three factors that together sustain tumor survival, motility, and drug resistance [64,65]. In addition, GO molecular function analysis showed that these genes are involved in phospholipase and lipase activator activity, as well as endopeptidase and protein-tyrosine kinase activities. Lipid mediators produced by phospholipases regulate several cellular processes, including proliferation, migration, invasion, and angiogenesis, which can promote tumorigenesis [66]. On the other hand, KEGG pathway enrichment highlighted signaling pathways such as AGE–RAGE signaling, VEGF signaling, lipid metabolism, and cell adhesion, which have been implicated in ovarian cancer progression and cell migration in previous studies.

Cancer cell migration is a multi-step process that relies on the coordinated activity of several organelles. Filopodia are slender, actin-rich protrusions that generally determine the direction of movement, whereas focal adhesions (FAs) are substantial, dynamic assemblies that couple the extracellular matrix to the actin cytoskeleton and mediate cell–substrate adhesion, a critical aspect of many migratory modes [67]. The assembly and turnover of FAs depend on a repertoire of structural and regulatory components, such as integrins. FA dynamics are crucial for cancer cell motility because actomyosin contractility at integrin-based FAs generates tensile forces, promotes rear retraction, and supports efficient cell migration [57,58]. In a study of vascular smooth muscle cell (VSMC) migration, treatment with the antioxidant NAC effectively inhibited migration. The results indicated that NAC reduced phosphorylation of focal adhesion kinase (FAK) and disrupted the assembly of focal adhesion complexes in treated VSMCs [68], which is consistent with the enrichment of migration-related pathways observed in our analysis. It is worth noting that the positive effects of antioxidant supplements, including cancer prevention and treatment support, stem from the idea that excessive levels of ROS cause oxidative damage to cellular macromolecules [69]. However, in *KRAS* and *BRAF*-driven lung cancer models, dietary NAC and vitamin E have been shown to reduce ROS and oxidative DNA damage in tumors. This reduction in oxidative stress suppressed *p53* activation, which then led to faster tumor growth and shorter survival. Importantly, these effects were not observed in tumors with inactive *p53*, suggesting that antioxidants may enable tumor cells to escape ROS-induced tumor suppressor pathways [70]. A study in hepatocellular carcinoma (HCC) also found that NAC and GSH enhanced HCC tumor formation and growth, along with a significant reduction in intracellular ROS levels [71]. Therefore, these observations suggest that the effects of antioxidants are highly context-dependent and are influenced by the type of compound, dose, and type of cancer [69].

A study demonstrated that epithelial ovarian carcinomas markedly upregulate the Receptor for Advanced Glycation-End Products (RAGE), a plasma membrane pattern recognition receptor that binds oxidized or glycation-modified ligands, at both mRNA and protein levels, and this over-expression rises with tumor stage and invasiveness [72]. Oral administration of NAC at 1 g L^−1^ for one week to Apolipoprotein E knock-out (*ApoE*^−^/^−^) mice suppressed RAGE itself, the inflammatory transcription factor nuclear-factor κB (NF-κB) p65 subunit, and the matrix-degrading enzymes matrix metalloproteinase-2 and -9 (*MMP2/9*), thereby disrupting the self-amplifying RAGE-to-NF-κB loop that also drives ovarian cancer dissemination [73]. The KEGG analysis also revealed that the VEGF signaling pathway is another crucial target for both compounds. VEGF signaling has been shown to facilitate tumor cell migration and invasion through potential mechanisms of dedifferentiation and epithelial–mesenchymal transition (EMT) [74]. NAC and AKG have been shown to directly impact tumor cell metastasis, as reported in other studies [45,73,74,75,76,77]. At the cellular level, NAC has been demonstrated to inhibit endothelial cell invasion and migration in vitro, likely due to its suppression of metalloproteinase activity [75]. Several studies have suggested that NAC may influence pathways associated with angiogenesis, including VEGF-related signaling [45]. Similarly, a study demonstrated that AKG significantly reduced extracellular signal-regulated kinases 1/2 (*ERK1/2*) phosphorylation in Saos-2 cells, a key driver of cell proliferation, migration, and metastasis in osteosarcoma. This reduction suppressed pro-metastatic factors such as transforming growth factor-beta (TGF-β) and VEGF, which are crucial for promoting cell migration, invasion, and angiogenesis [19]. The lipid and atherosclerosis pathway is one of the other crucial targets for both compounds. It clusters genes that govern exogenous fatty acid uptake (*CD36*, *LPL*, and *LDLR*), de novo lipogenesis (*FASN* and *SCD1*), lipid-peroxidation defense (*GPX4* and *APOA1/2*), and pro-inflammatory adhesion (VCAM-1 and ICAM-1); all are transcriptional outputs of sterol regulatory element-binding proteins (SREBPs) or NF-κB, which are constitutively active in HGSOC [78,79,80]. Taken together, our results suggest that a combination of NAC and AKG treatment is associated with reduced OVCAR3 cell survival and motility, in line with enrichment of apoptosis- and stress-related biological processes identified by network analysis.

To confirm the results of the KEGG and GO analyses, which indicated enrichment in migration-related pathways, a scratch assay was performed. The scratch assay provided additional phenotypic evidence consistent with reduced migratory behavior following combined treatment. Several studies have highlighted the anti-metastatic properties of both substances; disrupting the mentioned pathways with AKG [11] and NAC [45] might be crucial in decreasing the invasion and migration of ovarian cancer cells via different pathways and targeting several associated genes.

The results of the colony formation of OVCAR3 cells also showed that combined treatment with AKG and NAC resulted in fewer and more scattered colonies. This may suggest that the combined treatment affects cell proliferation and adherence, potentially influencing the cells’ ability to form organized colonies. In this regard, previous research indicated that the use of AA6, due to the increase in AKG levels, leads to a decrease in cell colonization by increasing the intracellular NO level [81]. Additionally, NAC significantly reduced cell motility in colorectal adenocarcinoma Caco-2 cells by increasing cell–cell and cell–matrix adhesions. This led to sparse monolayer colonies and multilayered growth in control cultures. Furthermore, NAC affected cell division by inhibiting tubulin polymerization, which disrupted the formation of the mitotic spindle and caused abnormal prophase characteristics in Caco-2 cells [56].

Our experimental validation indicates that combined AKG and NAC treatment produced the highest apoptosis rate in OVCAR3 cells, suggesting a stronger apoptotic phenotype under combination treatment. These findings are consistent with prior studies reporting that AKG and NAC can influence signaling contexts involving AKT, a critical regulator of cell survival and proliferation, in other cancer models [82]. Another study reported that NAC exerted anticancer effects in oral cancer through the EGFR/AKT/HBP1 signaling pathway [24]. In their study, NAC inhibited the EGFR/AKT pathway, often overactive in cancer cells, by reducing p-AKT. This reduction inhibited cell growth and promoted apoptosis. Furthermore, they claimed that NAC induced the transcription of pro-apoptotic genes such as Bcl-2-associated X protein (Bax) and Fas ligand (FasL), which in turn activated caspase-3, a key enzyme responsible for executing apoptosis by cleaving cellular components [24].

Collectively, prior studies have implicated the PI3K/AKT pathway as an important regulator of cell survival and apoptotic balance in cancer cells [83]. The PI3K/AKT signaling cascade is widely recognized as a central intracellular pathway involved in the regulation of cell proliferation and apoptosis [83]. AKT (Protein kinase B), a downstream effector of PI3K, has been reported to be associated with enhanced cell survival and growth through modulation of multiple downstream effectors, including apoptosis-related proteins [84]. AKT activation is commonly associated with phosphorylation events triggered by growth factors and extracellular stimuli [83]. Based on prior studies, *AKT* has been proposed as a potential molecular node through which NAC and AKG may exert growth-modulatory effects (Supplementary Materials, Sections S1 and S2). However, direct pathway-level validation was beyond the scope of the present study.

Notably, our findings on NAC’s antiproliferative effects in OVCAR3 align with previous reports [56], which demonstrated that mid-to-high NAC concentrations (0.5–10 mM, Sigma-Aldrich) exerted a dose-dependent reduction in proliferation without affecting cell viability and further impaired motility in Caco-2 colorectal adenocarcinoma cells by increasing cell–cell and cell–matrix adhesions, accompanied by disruption of mitotic spindle formation. In our study, NAC (10 mM, BioBasic) in combination with 100 µM AKG was associated with a more pronounced apoptotic phenotype and reduced migratory behavior compared with single-agent treatment. This suggests that while NAC alone has known antiproliferative activity, its combination with AKG may enhance these cellular effects in the ovarian cancer context. In line with these observations, our PPI network analysis identified *AKT1* and *CASP3* as central hub genes with among the highest MCC scores, highlighting their pivotal positions within the network. Exploratory protein-level observations revealed treatment-associated changes in *AKT1* and *CASP3*, consistent with patterns reported in prior studies using OVCAR3 cells.

Taken together, the network pharmacology results and in vitro observations, in conjunction with previously published studies discussed above, were integrated into a conceptual, hypothesis-generating schematic (Supplementary Materials, Figure S3) to illustrate potential relationships among NAC, AKG, and apoptosis- and survival-associated signaling processes in OVCAR3 cells.

## 5. Limitations

This study has some limitations that should be considered when interpreting the findings. First, all experimental validations were conducted using the OVCAR3 cell line. Future studies incorporating additional high-grade serous ovarian cancer models and patient-derived samples will be important to assess the broader applicability of the observed effects. Second, the experimental work was performed under in vitro conditions. While in vitro assays provide controlled and reproducible systems to examine cellular responses, they do not recapitulate key aspects of the in vivo tumor context, including stromal interactions, immune modulation, metabolic constraints, and pharmacokinetic variability. As such, the phenotypic effects observed here may not directly translate to in vivo conditions. Third, these findings should be interpreted as proof-of-concept observations rather than direct indicators of clinical efficacy. Finally, although complementary computational and molecular analyses supported the phenotypic findings, these approaches were exploratory and not intended to establish definitive causal relationships. Accordingly, further studies with expanded biological replication, quantitative validation, and in vivo modeling will be required to strengthen the biological and translational relevance of the present observations.

## 6. Conclusions

In conclusion, this study investigated the effects of combined AKG and NAC treatment on OVCAR3 cells using both in vitro experiments and network pharmacology analysis. The network pharmacology approach identified key target genes, highlighting their relevance to ovarian cancer progression. The results demonstrate that combined NAC and AKG treatment induces more pronounced phenotypic effects in OVCAR3 ovarian cancer cells than either compound alone under in vitro conditions. Specifically, the combination was associated with reduced cell viability, impaired migratory behavior, and altered clonogenic capacity, suggesting an overall attenuation of cellular traits commonly linked to tumor progression.

Overall, this work provides a rationale for further investigation of NAC and AKG in additional ovarian cancer models. Future studies incorporating in vivo validation, broader dose–response analyses, and expanded phenotypic and metabolic profiling will be necessary to better define the biological significance and potential translational relevance of this combinatorial approach.

## Figures and Tables

**Table 1:** Hub genes and their biological functions.

Gene Symbol	Gene Name	MCC Score	Function
*AKT1*	RAC-alpha serine/threonine-protein kinase	111,814	*AKT1* is one of three homologous serine/threonine protein kinases—*AKT1*, *AKT2*, and *AKT3*—collectively designated as the AKT kinases. It is integral to the regulation of various cellular functions, such as metabolism, proliferation, cell survival, growth, and angiogenesis [29]. In ovarian cancer/HGSOC, aberrant PI3K/AKT signaling promotes cell survival, proliferation, and therapy resistance, making *AKT1* a key node in survival–apoptosis regulation [30].
*CASP3*	Caspase-3	109,702	Thiol proteases serve as key caspase effectors, playing a pivotal role during the execution phase of apoptosis [31,32]. In ovarian cancer, caspase-3 activation is a central execution step of apoptosis and directly reflects engagement of death pathways targeted by anticancer interventions [33].
*HSP90AA1*	Heat shock protein HSP 90-alpha	106,549	A molecular chaperone that facilitates the proper folding, structural stability, and regulation of specific target proteins—such as those involved in cell cycle control and signal transduction. Its activity is linked to an ATPase cycle, which is crucial for its chaperone function. This cycle likely causes conformational changes in client proteins, leading to their activation. It also interacts dynamically with various co-chaperones that influence substrate recognition, ATPase activity, and overall chaperone function [34,35,36,37,38,39]. In ovarian cancer, *HSP90* supports the stability of multiple oncogenic client proteins such as kinases in PI3K/AKT and MAPK pathways, linking it to tumor cell survival and stress adaptation [40].
*SRC*	Proto-oncogene tyrosine-protein kinase Src	101,893	A non-receptor protein tyrosine kinase that becomes activated upon engagement of various cellular receptors, including immune response receptors, integrins, other adhesion receptors, receptor tyrosine kinases, G protein-coupled receptors, and cytokine receptors [41]. In ovarian cancer, SRC-family signaling contributes to adhesion, migration, and invasion through focal adhesion/FAK and downstream PI3K/AKT and MAPK cascades [42].
*GSK3B*	Glycogen synthase kinase-3 beta	100,128	Negatively modulates the extrinsic apoptotic signaling pathway through death domain receptors. It facilitates the assembly of an anti-apoptotic complex comprising *DDX3X*, *BRIC2*, and *GSK3B* at death receptors such as *TNFRSF10B*. This anti-apoptotic effect is most pronounced under conditions of weak apoptotic stimuli and can be overridden by more robust activating signals [43]. In ovarian cancer, GSK3B intersects with survival and EMT-related programs and can modulate apoptosis-related signaling downstream of PI3K/AKT [44].
*CDC42*	Cell division control protein 42	99,582	It plays a role in regulating cell migration [45]. In ovarian cancer, *CDC42* regulates cytoskeletal remodeling and directional migration, consistent with the migration/adhesion pathways highlighted in this study [46].
*HRAS*	GTPase HRas	92,244	Plays a role in activating Ras protein-mediated signal transduction [47]. In ovarian cancer, RAS signaling feeds into MAPK/ERK and PI3K/AKT pathways, influencing proliferation and survival programs [48].
*MMP9*	Matrix metalloproteinase-9	90,776	Matrix metalloproteinases are crucial for mediating local extracellular matrix proteolysis and facilitating leukocyte migration [49]. In ovarian cancer, *MMP9* facilitates extracellular matrix remodeling and invasion, linking this hub gene to migration and metastatic potential [50].
*RAF1*	RAF proto-oncogene serine/threonine-protein kinase	56,524	This serine/threonine protein kinase serves as a regulatory intermediary linking membrane-associated Ras GTPases to the MAPK/ERK signaling cascade. It functions as a critical switch that influences cell fate decisions, including proliferation, differentiation, apoptosis, survival, and oncogenic transformation [51]. In ovarian cancer, *RAF1* transduces signals through the MAPK/ERK pathway and contributes to proliferation and survival signaling cross-talk [52].
*LCK*	Tyrosine-protein kinase Lck	42,864	This non-receptor tyrosine kinase is crucial for the selection and maturation of developing T-cells in the thymus and the functional activity of mature T-cells. It is a key mediator in the signal transduction pathways associated with the T-cell antigen receptor (TCR) [53]. In ovarian cancer microenvironments, LCK-related signaling can reflect immune-associated pathway activity and may intersect with kinase networks identified in the PPI analysis [54].

## Data Availability

The original contributions presented in this study are included in the article/Supplementary Materials. Further inquiries can be directed to the corresponding authors.

## Supplementary Materials

The following supporting information can be downloaded at https://www.mdpi.com/article/10.3390/cells15030281/s1, Section S1: Supplementary Methods; Section S2: Supplementary Results; Section S3: Conceptual model and hypothesis-generating framework. Figure S1: Association of *CASP3* and *AKT1* transcript levels with progression-free survival in ovarian cancer; Figure S2: Expression levels of p-AKT1/AKT1 and cleaved/pro-caspase-3 in OVCAR3 cells after 24 h treatment with a combination of NAC and AKG; Figure S3: Schematic model of the proposed NAC–AKG–AKT–CASP3 axis in OVCAR3 cells; Table S1: Networkpharmacology network analysis data; Table S2: Hub Scores; Table S3: Synergistic Analysis.
