# Supplementary material for: Effects of N-Acetylcysteine and Alpha-Ketoglutarate on OVCAR3 Ovarian Cancer Cells: Insights from Integrative Bioinformatics and Experimental Validation
Source: Cells. 2026 Feb 2;15(3):281. doi: 10.3390/cells15030281 (PMC12897456; doi:10.3390/cells15030281)
Supplement: Supplementary file 1 [file cells-15-00281-s001.zip › Supplementary materials.pdf]

Effects of N-Acetylcysteine and Alpha-Ketoglutarate on OVCAR3 Ovarian Cancer Cells: Insights from Integrative Bioinformatics and Experimental Validation

S1. Supplementary Methods

S1.1. Differential expression and prognostic significance of CASP3 and AKT1 in ovarian cancer

To evaluate the prognostic significance of CASP3 and AKT1, the two hub genes with the highest MCC scores in the PPI network, their differential expressions were analyzed with GEPIA (http://gepia.cancer pku.cn), which integrates harmonized TCGA and GTEx RNA seq data. Expression profiles from 426 ovarian carcinoma specimens (TCGA OV) and 88 normal ovarian tissues (GTEx) were compared; genes with $|\log_2$ fold change$| > 1$ and $P < 0.01$ (Student's t-test) were considered significant. The prognostic value of the CASP3:AKT1 expression ratio was subsequently assessed in the TCGA cohort using the Kaplan–Meier Plotter (http://kmplot.com). Patients were stratified at the median ratio, and progression-free survival (PFS) was analyzed with the log-rank test ($P < 0.05$). Hazard ratios (HRs) and their 95% confidence intervals (CIs) were estimated using a univariate Cox proportional hazards model. The CI represents the range within which the accurate HR is expected to lie with 95 % confidence, thereby providing a measure of precision for the survival risk estimate. The numbers at risk were automatically displayed beneath each survival curve. Based on their consistent prioritization in the network analysis, CASP3 and AKT1 were further examined at the protein level in an exploratory manner using an in vitro OVCAR3 cell model.

S.1.2. Western blot analysis

To provide additional observational context to the transcriptomic findings, protein-level expression of CASP3 and AKT1 was assessed in OVCAR3 cells following treatment with NAC and AKG. OVCAR3 cells were plated in 6-well plates and subsequently treated with 10 mM NAC, 100 μM AKG, and a combination of both compounds for 24 hours. Following this treatment, the cells were homogenized in 500 μL of lysis buffer (20 mM Tris-HCl pH 8.0, 2 mM EDTA, 150 mM NaCl, 0.5% sodium deoxycholate, 0.1% SDS, and 1% NP-40, supplemented with protease inhibitor cocktail) for 10 minutes and then centrifuged at 12,000 rpm for 10 minutes to isolate the supernatant. The protein concentration in the samples was determined using the Bradford assay with BSA as the standard, after which the proteins were separated using sodium dodecyl sulfate–polyacrylamide gel electrophoresis (SDS-PAGE) and transferred to polyvinylidene difluoride (PVDF) membranes. The PVDF membranes were blocked at room temperature for 1 hour with 5% non-fat skimmed milk in PBS-T (PBS containing 0.1% Tween-20). The membranes were incubated overnight at 4°C with specific primary antibodies (pro-caspase-3 [9662, 1:1000, CST], cleaved caspase-3 [9661, 1:1000, CST], AKT1 [sc-5298, 1:200, Santa Cruz], phosphorylated AKT (Ser473) [9271, 1:1000, CST], and β-actin [sc-47778, 1:300, Santa Cruz]). After this, the PVDF membranes were washed three times with PBS-T for 5 minutes each. The blots were then exposed to HRP-conjugated secondary antibodies (anti-rabbit IgG-HRP (sc-2357, Santa Cruz Biotechnology; 1:1000) and m-IgGκ BP-HRP (sc-516102, Santa Cruz Biotechnology; 1:1000) and incubated at room temperature for 1 hour on a shaker. The chemiluminescent signal was developed using an enhanced chemiluminescence (ECL) detection kit and recorded on photographic films in a darkroom using standard developing and fixing solutions. After film development, the protein bands were analyzed using ImageJ software.

S2. Supplementary Results

S2.1. High CASP3:AKT1 expression ratio is associated with shorter PFS in patients with ovarian cancer

Based on their consistent prioritization in the network pharmacology analysis, CASP3 and AKT1 were further examined at the transcriptomic level using public ovarian cancer datasets. Transcriptomic profiling of the TCGA OV dataset showed that both CASP3 and AKT1 were significantly more highly expressed in tumor tissue compared to normal ovaries, suggesting their involvement in the malignant transcriptome (Figure S1A). Furthermore, in the TCGA ovarian cancer cohort (n = 1001), patients were divided at the median CASP3 : AKT1 mRNA expression into low-ratio (n = 749) and high-ratio (n = 252) groups. Kaplan–Meier analysis demonstrated an early and persistent separation of survival curves, with the high-ratio group exhibiting shorter progression-free survival compared to the low-ratio group (log-rank $p = 9 \times 10^{-4}$). Univariate Cox regression yielded a hazard ratio of 1.31 (95 % CI 1.12–1.54), indicating an increased risk of progression or death associated with the high-ratio group. After 50 months of follow-up, 52 patients in the low-ratio group were still progression free, whereas only 17 remained progressions free in the high-ratio group (Figure S1B). Collectively, these observations indicate an association between the CASP3:AKT1 mRNA expression ratio and PFS in the TCGA OV cohort and support its consideration as an exploratory, hypothesis-generating transcriptomic marker.

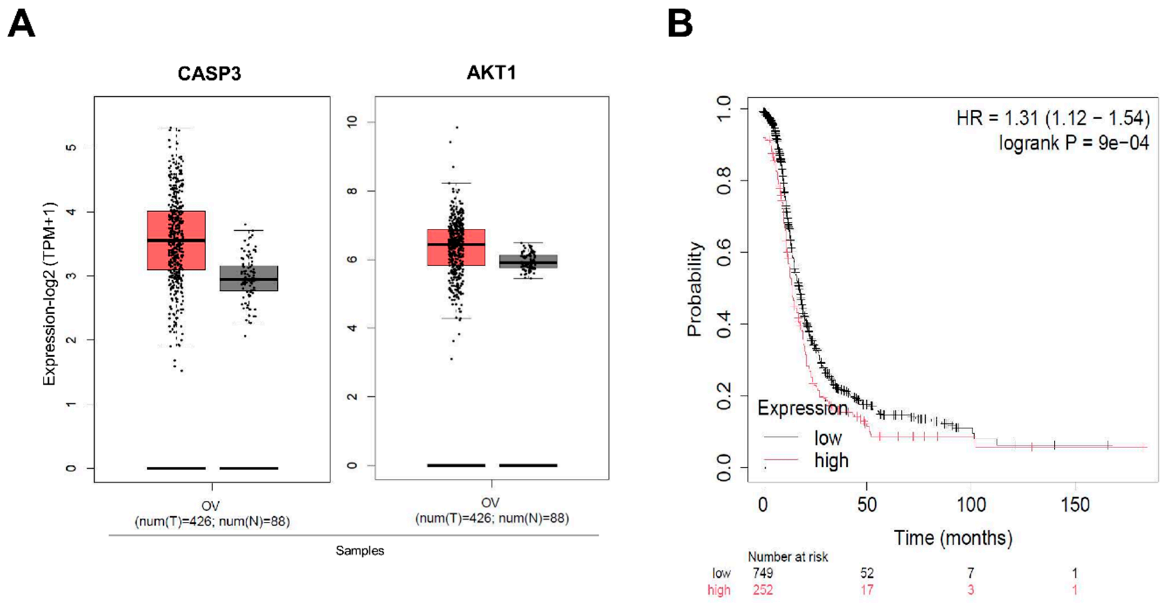

Figure S1. Association of CASP3 and AKT1 transcript levels with progression-free survival in ovarian cancer. (A) CASP3 and AKT1 expression levels (log2[TPM+1]) are significantly higher in ovarian tumor tissue (pink, n = 426) than in normal ovary (gray, n = 88) based on TCGA and GTEx transcriptomic data. (B) Progression-free survival (PFS) in the TCGA OV cohort (n = 1,001) stratified by the median CASP3:AKT1 ratio. Patients with a high ratio showed shorter PFS compared to those with a low ratio (log-rank $p = 9 \times 10^{-4}$). Univariate Cox regression yielded a hazard ratio (HR) of 1.31 with a 95 % confidence interval (CI) of 1.12–1.54, reflecting an association between a higher ratio and increased risk of progression or death. The number at risk is shown below the plot.

S.2.2. Exploratory Western blot observations of AKT1 and caspase-3 in NAC- and AKG-treated OVCAR3 cells

To provide additional observational context for the transcriptomic findings, Western blot analysis was performed to examine protein expression patterns of AKT1, p-AKT1, pro-caspase-3, and cleaved caspase-3 in OVCAR3 cells following treatment with NAC, AKG, or their combination (Figure S2A). Total AKT1 levels showed minimal variation across treatment conditions, whereas treatment-associated

differences were observed in p-AKT1/AKT1 and cleaved/pro-caspase-3 ratios. The combined NAC and AKG treatment were associated with more pronounced changes in these ratios compared with single-

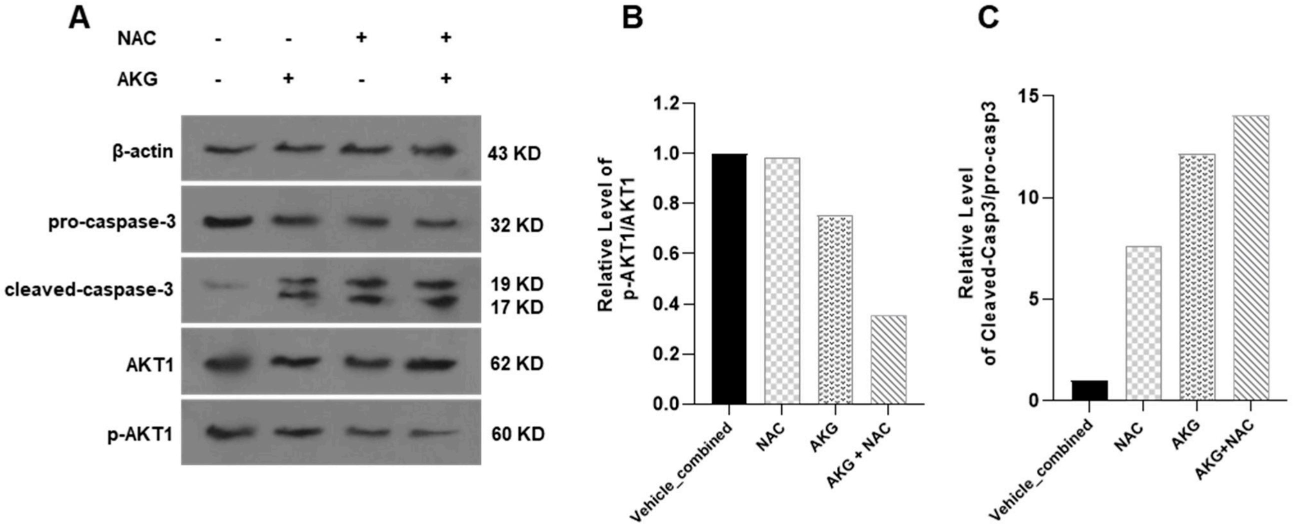

agent treatments (Figure S2B,C). These observations are presented without statistical inference (n=1).

Figure S2. Expression levels of p-AKT1/AKT1 and cleaved/pro-caspase-3 in OVCAR3 cells after 24 hr treatment with a combination of NAC and AKG. (A) Representative immunoblots. (B, C) Representative densitometry of p-AKT1/AKT1 and cleaved-caspase-3/pro-caspase-3. Western blotting was performed once (single experiment; n = 1); therefore, no inferential statistics were applied.

S3. Conceptual model and hypothesis-generating framework

This figure provides a conceptual and hypothesis-generating framework that integrates the observations of the present study with relevant literature, as discussed in the Discussion section.

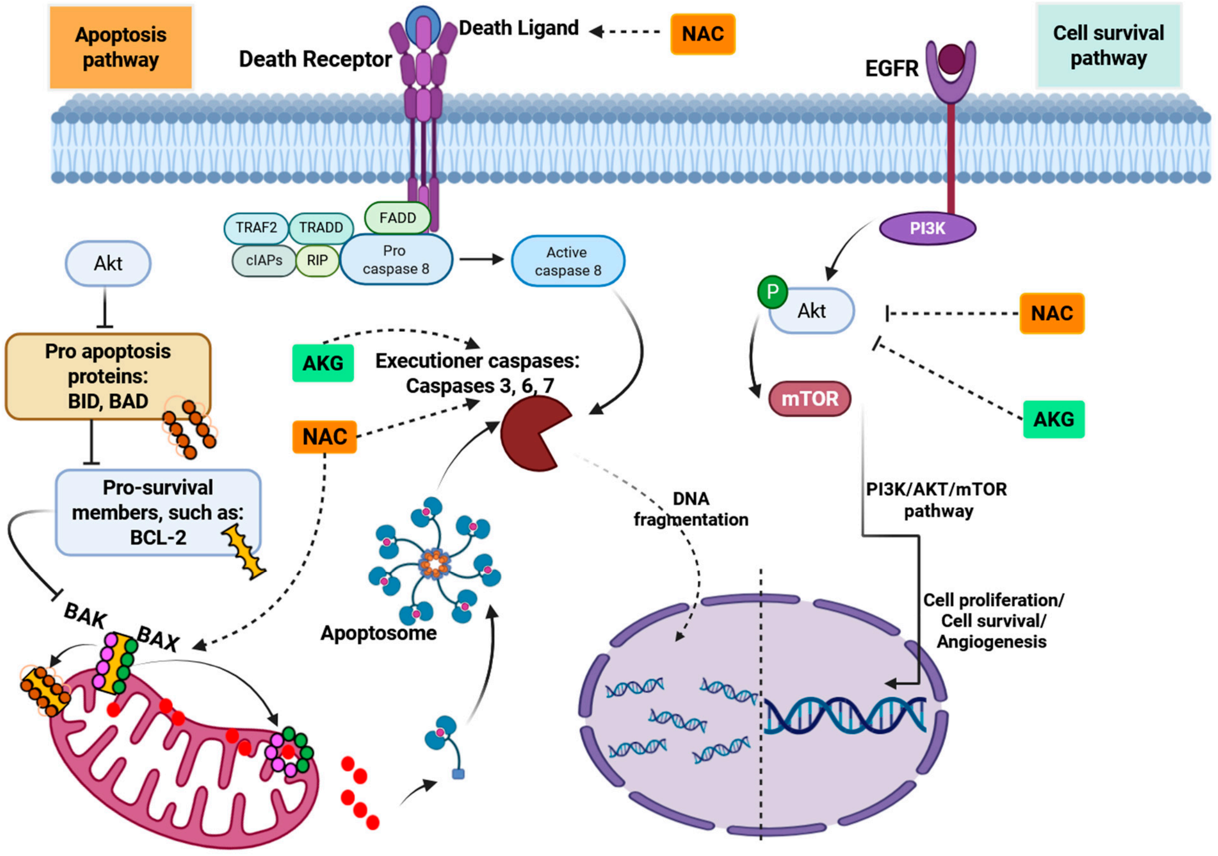

Figure S3. Schematic model of the proposed NAC–AKG–AKT–CASP3 axis in OVCAR3 cells. The diagram presents a hypothesis-driven model of how NAC and AKG may influence survival and apoptotic signaling, integrating our network pharmacology predictions with exploratory in vitro observations. NAC and AKG are depicted as potential modulators of the PI3K/AKT/mTOR survival pathway, consistent with the reduction in p-AKT1 observed under combination treatment conditions. Reduced AKT signaling could alleviate anti-apoptotic signaling and thereby may facilitate activation of the caspase cascade, including executioner caspases such as CASP3, in line with the increase in cleaved caspase-3 detected in our assay. The schematic also illustrates death receptor–associated initiator caspase-8 activation and mitochondrial amplification steps (BAX/BAK-mediated permeabilization, cytochrome c release, and apoptosome formation) that may contribute to executioner caspase activation and downstream DNA fragmentation. Solid arrows represent previously reported pathway relationships, whereas dashed arrows indicate putative links inferred from the present study and supporting literature.
